# Whole exome sequencing identified a homozygous novel variant in *CEP290* gene causes Meckel syndrome

**DOI:** 10.1111/jcmm.14887

**Published:** 2019-12-15

**Authors:** Rui Zhang, Shaoyun Chen, Peng Han, Fangfang Chen, Shan Kuang, Zhuo Meng, Junnian Liu, Ruliang Sun, Zhiwei Wang, Xiaohong He, Yong Li, Yuanning Guan, Zhengfang Yue, Chen Li, Subrata Kumar Dey, Yuanfang Zhu, Santasree Banerjee

**Affiliations:** ^1^ Division of Maternal‐Fetal Medicine Bao'an Women and Children's Hospital Jinan University Shenzhen China; ^2^ BGI‐Qingdao BGI‐Shenzhen Qingdao China; ^3^ China National GeneBank BGI‐Shenzhen Shenzhen China; ^4^ Department of Pathology Bao'an Maternity and Child Health Hospital Shenzhen China; ^5^ BGI‐Shenzhen Beishan Industrial Zone Shenzhen China; ^6^ BGI‐Genomics BGI‐Shenzhen Shenzhen China; ^7^ Department of Cell Biology and Medical Genetics School of Medicine Zhejiang University Hangzhou China; ^8^ Department of Biotechnology Centre for Genetic Studies School of Biotechnology and Biological Sciences Maulana Abul Kalam Azad University of Technology (Formerly West Bengal University of Technology) Kolkata India; ^9^ Brainware University Barasat India

**Keywords:** *CEP290* gene, homozygous, *loss‐of‐function*, Meckel syndrome, novel variant

## Abstract

Meckel syndrome (MKS) is a pre‐ or perinatal multisystemic ciliopathic lethal disorder with an autosomal recessive mode of inheritance. Meckel syndrome is usually manifested with meningo‐occipital encephalocele, polycystic kidney dysplasia, postaxial polydactyly and hepatobiliary ductal plate malformation. Germline variants in *CEP290* cause MKS4. In this study, we investigated a 35‐years‐old Chinese female who was 17+1 weeks pregnant. She had a history of adverse pregnancy of having foetus with multiple malformations. We performed ultrasonography and identified the foetus with occipital meningoencephalocele and enlarged cystic dysplastic kidneys. So, she decided to terminate her pregnancy and further genetic molecular analysis was performed. We identified the aborted foetus without postaxial polydactyly. Histological examination of foetal kidney showed cysts in kidney and thinning of the renal cortex with glomerular atrophy. Whole exome sequencing identified a novel homozygous variant (c.2144T>G; p.L715^*^) in exon 21 of the *CEP290* in the foetus. Sanger sequencing confirmed that both the parents of the foetus were carrying this variant in a heterozygous state. This variant was not identified in two elder sisters of the foetus as well as in the 100 healthy individuals. Western blot analysis showed that this variant leads to the formation of truncated CEP290 protein with the molecular weight of 84 KD compared with the wild‐type CEP290 protein of 290 KD. Hence, it is a *loss‐of‐function* variant. We also found that the mutant cilium appears longer in length than the wild‐type cilium. Our present study reported the first variant of *CEP290* associated with MKS4 in Chinese population.

## 1 INTRODUCTION

Meckel syndrome type 4 (MKS4) [MIM# 611134] is a pre‐ or perinatal lethal ciliopathic disorder with an autosomal recessive mode of inheritance.^1^ Meckel syndrome is manifested with occipital meningoencephalocele, postaxial polydactyly, multicystic kidney dysplasia and hepatobiliary ductal plate malformation.^2^ Meckel syndrome patients are usually died before or soon after birth. In addition, the incidence of MKS is 1 in 140 000 live birth worldwide.^3^ In Finnish and Belgian population, the occurrence of MKS is more than the other populations.^3^ Genetically, MKS is extremely heterogenous and associated with germline variants of a group of genes.^4^ Moreover, MKS has been reported to be caused by the germline variants of eight genes (*CEP290, MKS1, B9D1, B9D2, CC2D2A, RPGRIP1L, TMEM67, TMEM216*).^4^ These eight genes and their encoded proteins are playing the key role in the formation of cilia.^5^ Structurally, cilia are located on the cellular surface and maintaining the structure and function of a group of cells, namely brain cells, kidney cells and liver cells.^6^ Cilia are also involved in transmitting signals among adjacent cells.^6^, ^7^ So, germline variants of any of these eight genes exert effects on the structure and function of cilia which finally results into MKS.

Germline variants of *CEP290* gene cause MKS4. *CEP290* gene is located in the long arm (q) of chromosome 12.^8^ The *CEP290* gene has 54 exons and encodes CEP290 (centrosomal protein of 290 kD) protein consisting of 2479 amino acids.^8^ Till date, more than 100 variants of *CEP290* has been reported which mostly causes Leber congenital amaurosis 10 [MIM# 611755] and Joubert syndrome 5 [MIM# 610188]. In contrast, variants of *CEP290* have been reported to cause MKS 4 [MIM# 611134] in very few cases. Among those reported variants of *CEP290*, most of them are classified as *loss‐of‐function* (non‐sense, frameshift or splice‐site variants) variants.^2^

In this study, we investigated a 35‐years‐old Chinese female who was 17+1 weeks pregnant (gravida 6, para 2). She had a history of adverse pregnancy of having foetus with multiple malformations. We performed ultrasonography and identified the foetus with all classic MKS symptoms, that is occipital meningoencephalocele, enlarged cystic dysplastic kidneys. So, she decided to terminate her pregnancy and further genetic molecular analysis was performed. We found the aborted foetus without postaxial polydactyly. Histological examination of the foetal kidney showed cysts in kidney and thinning of renal cortex with glomerular atrophy. The histology of the foetal liver is completely normal without hepatobiliary ductal plate malformation. Karyotype analysis and chromosomal microarray found no chromosomal abnormalities in the foetus. Genomic DNA has been extracted from the skin of the foetus. Whole exome sequencing identified a novel homozygous variant (c.2144T>G; p.L715^*^) in exon 21 of the *CEP290* gene in the foetus. Sanger sequencing confirmed that both the parents of the foetus are heterozygous for this variant. Our present study identified the first variant in *CEP290* gene associated with MKS in Chinese population. In this study, we also emphasize the significance of whole exome sequencing for identifying candidate variant in the MKS patients with *CEP290* variants.

## 2 MATERIALS AND METHODS

### 2.1 Patients and families

**Figure 1:**
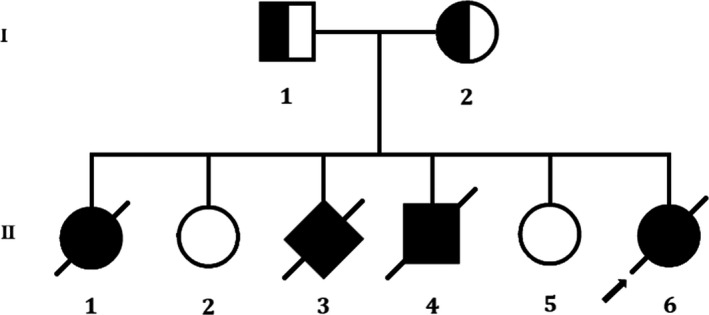
Pedigree of the described non‐consanguineous Chinese family with MKS. Squares and circles denoted males and females respectively. Individuals labelled with a solidus were deceased. Roman numerals indicate generations. Arrow indicates the proband (II‐6)

Here, a Han Chinese family with Meckel syndrome was enrolled in the Division of Maternal‐Fetal Medicine, Bao'an Women and Children's Hospital, Jinan University, Shenzhen, China (Figure 1). Normal kidney tissue was collected for experiment. The study was approved by the ethics committee of the Bao'an Women and Children's Hospital, Jinan University, Shenzhen, China, in accordance with the recommendations of the Declaration of Helsinki. We obtained written informed consent from all the participant of this study.

### 2.2 Karyotype and chromosomal microarray analyses

In order to analyse the structure of all the chromosomes in the foetus, we performed standard G‐banding karyotyping. Next, in order to confirm the presence of copy number variations (CNV) in the foetus, chromosome microarray analysis was performed using a CytoScan HD array (Affymetrix), according to the manufacturer's protocols (Affymetrix). Chromosome Analysis Suite software version 1.2.2 were used for analysing the data. The reporting threshold of copy number was set at 10 kb with marker count at ≥50.^9^

### 2.3 Whole exome sequencing and identification of variants

Genomic DNA of foetus was extracted from the skin of the foetus according to the manufacturer's instructions. Genomic DNA of the foetus was subjected to whole exome sequencing. Agilent SureSelect version 4 (Agilent Technologies) was used to capture the sequences. We use Illumina HighSeq 4000 platform to sequence the enriched library. Next, we use Burrows‐Wheeler Aligner software (version 0.59) for aligning the sequencing reads with GRCh37.p10. After that, local alignment and recalibration of base quality of the Burrows‐Wheeler aligned reads was performed by the GATK Indel Realigner and the GATK Base Recalibrator, respectively (broadinstitute.org/). Then, GATK Unified Genotyper (broadinstitute.org/) was used for identifying single‐nucleotide variants (SNVs) and small insertions or deletions (InDels). Finally, the identified variants were annotated with the Consensus Coding Sequences Database (20130630) at the National Center for Biotechnology Information (NCBI). The quality control of whole exome sequencing has been illustrated in Table 1.

**Table 1:** Quality control data of whole exome sequencing

Total	
Raw reads (All reads)	415 859 158
QC Fail reads	0
Raw data (Mb)	20 792.96
Paired reads	415 859 158
Mapped reads	413 522 700
Fraction of mapped reads	99.44%
Mapped data (Mb)	20 676.13
Fraction of mapped data (Mb)	99.44%
Properly paired	403 742 452
Fraction of properly paired	97.09%
Read and mate paired	412 466 178
Fraction of read and mate paired	99.18%
Singletons	1 056 522
Read and mate map to different chromosome	7 602 644
Read1	207 929 579
Read2	207 929 579
Read1 (rmdup)	118 938 717
Read2 (rmdup)	118 900 003
forward strand reads	206 779 635
backward strand reads	206 743 065
PCR duplicate reads	175 683 980
Fraction of PCR duplicate reads	42.48%
Map quality cutoff value	20
Map quality above cutoff reads	376 001 764
Fraction of map Q reads in all reads	90.42%
Fraction of map Q reads in mapped reads	90.93%
Target	
Target reads	232 283 481
Fraction of target reads in all reads	55.86%
Fraction of target reads in mapped reads	56.17%
Target data (Mb)	10 652.28
Target data Rmdup (Mb)	5805.82
Fraction of target data in all data	51.23%
Fraction of target data in mapped data	51.52%
Len of region	58 682 415
Average depth	181.52
Average depth (rmdup)	98.94
Coverage (>0x)	99.76%
Coverage (≥4x)	99.63%
Coverage (≥10x)	99.20%
Coverage (≥30x)	95.99%
Coverage (≥100x)	68.77%
Target region count	199 824
Region covered >0x	199 275
Fraction region covered >0x	99.73%
Fraction region covered ≥4x	99.58%
Fraction region covered ≥10x	99.19%
Fraction region covered ≥30x	96.62%
Fraction region covered ≥100x	69.26%
Flank	
Flank size	200
Len of region (not include target region)	70 656 846
Average depth	36.45
Flank reads	75 095 626
Fraction of flank reads in all reads	18.06%
Fraction of flank reads in mapped reads	18.16%
Flank data (Mb)	2575.32
Fraction of flank data in all data	12.39%
Fraction of flank data in mapped data	12.46%
Coverage (>0x)	96.87%
Coverage (≥4x)	83.23%
Coverage (≥10x)	60.81%
Coverage (≥30x)	35.09%
Coverage (≥100x)	9.14%

**Figure 2:**
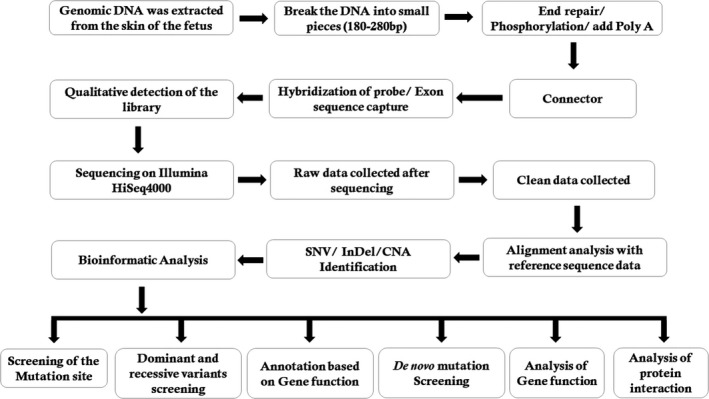
Schematic presentation of the detailed data interpretation pipeline

During interpretation and analysis of the data, we selected the variations if their minor allele frequencies are less than 0.05 in dbSNP (https://www.ncbi.nlm.nih.gov/SNP), HapMap (https://www.genome.gov/international-hapmap-project), 1000 Genomes Project (http://www.internationalgenome.org) and BGI database with ~50 000 Chinese Han samples. We also classified the identified variations into pathogenic, likely pathogenic, VUS, likely benign and benign groups according to the variant interpretation guidelines of American College of Medical Genetics and Genomics (ACMG).^10^ Lastly, we also analysed the remaining variations in the foetus as well as in his unaffected parents with the reference of the OMIM (https://www.omim.org) and other published literature. The detailed and comprehensive variant interpretation and analysis pipeline is schematically presented in Figure 2.

### 2.4 Sanger sequencing

In order to validate the identified variants by whole exome sequencing, we performed Sanger sequencing. Designing of primer pairs for the candidate loci has been done based on the reference genomic sequences of the Human Genome from GenBank in NCBI. Primer pairs were synthesized by Invitrogen, Shanghai, China. Polymerase chain reaction (PCR) was performed with an ABI 9700 Thermal Cycler and next, directly sequenced the PCR products by an ABI PRISM 3730 automated sequencer (Applied Biosystems). Analysis of sequencing data has been done by DNASTAR SeqMan (DNASTAR).

Whole exome sequencing identified the novel homozygous variant which was validated by Sanger sequencing using the following primers: F1 5′‐AGGCGATGCGGCGGTTTCTAGC‐3′, R1 5′‐GGCCGCGGCGATCGGCCGTG‐3′. The reference sequence NM_025114.3 of *CEP290* was used.

### 2.5 In vitro functional analysis of the novel variant by western blot

Foetal kidney tissue was rapidly frozen in liquid nitrogen and stored in ultra‐low temperature freezer. The RIPA lysis buffer (protease inhibitor: PMSF: lysis buffer = 1:2:200) was used to homogenize the foetal kidney tissue by tissue homogenizer (speed: 6.0 m/s, adapter: quickprep, time: 40 seconds). Protein samples were immediately frozen and centrifuged (4℃, 12 000*g*, 15 minutes), which were denatured (100°C, 5 minutes) and stored at −80℃. The protein concentration was detected with the BCA Assay Kit (Pierce). It is worth to be noting that the protein extraction process needs to be completed on ice. Equal amounts of protein per sample were separated by sodium dodecyl sulphate‐polyacrylamide gel electrophoresis (SDS‐PAGE) and then the proteins on the gel were transferred to the polyvinylidene fluoride (PVDF) membranes. The membranes were blocked with 5% bovine serum albumin (BSA, Sigma‐Aldrich) blocking solution for 1 hour at room temperature, and then were incubated in the primary antibody dilution overnight at 4°C. After washing three times with Tris‐buffered saline with 1% Tween20 (TBS‐T), the membranes were incubated in the secondary antibody dilution for 1 hour at room temperature in the dark. Finally, the protein bands were visualized with an Odyssey CLx imaging system (LI‐COR). All experiments were carried out independently at least three times. The protein level of *GAPDH* was used as the endogenous control. Western blot analysis was performed with the following antibodies: CEP290 (1:100, Santa Cruz Biotechnology, sc‐390462), *GAPDH* (1:10 000, Abcam, ab181602).

### 2.6 Immunofluorescence study of human kidney tissues

Foetal kidney tissue and also normal kidney tissues were fixed in 4% paraformaldehyde overnight and embedded in 4 μm thick paraffin. The paraffin block containing kidney tissue was cut into thin slices and baked them dry (60°C, 2 hours). Next, de‐paraffinization was performed (xylene I, 20 minutes; xylene II, 20 minutes; 100% ethyl alcohol, 5 minutes; 95% ethyl alcohol, 5 minutes; 70% ethyl alcohol, 5 minutes; double steamed water, 5 minutes/3 times; 3% H_2_O_2_, 5 minutes; double steamed water, 5 minutes/2 times; 0.3% triton X‐100, 10 minutes) and then boiled in citrate buffer (for antigen retrieval) in pH 6.0 (2 × 5 minutes microwave at 900 W). Blocking of non‐specific binding sites has been done by 5% BSA at room temperature for 1 hour and followed by incubation in 1% BSA buffer overnight at 4°C with appropriate antibodies (CEP290: sc‐390462, 1:50; ARL13B: 17711‐1‐AP, 1:50). Alexa Fluor 488 (1:400, # A32723) or Alexa Fluor 488 (1:400, # A32731) was added for 1 hour at 37°C. Then, the glass slides were rinsed in PBS (5 minutes/time, three times) and coverslips were fixed on glass slides with fluoroshield mounting medium (containing DAPI). Confocal images were taken using a scanning microscope system (Yokogawa CSU‐X1 spinning disk scanner coupled to a Zeiss Axio Imager Z2 inverted microscope and controlled by Zen Blue software).

### 2.7 In silico analysis

The variant identified in the foetus by whole exome sequencing was analysed by Mutation Taster (http://mutationtaster.org/).^11^

## 3 RESULTS

### 3.1 Human subjects

In this present study, we investigated a 35‐years‐old pregnant Chinese woman who had a history of adverse pregnancy (Figure 1). This Chinese family is a truly non‐consanguineous. She is 17+1‐week pregnant (gravida 6, para 2). It is her 6th pregnancy and she had successful deliveries of two girl children from her previous five pregnancies. During her 6th pregnancy, she went to our hospital for routine B‐ultrasound examination and we identified her foetus with multiple malformations.

During consultation, it has been revealed that she had a history of adverse pregnancy for three times among her last five pregnancies. In 2006, during her first pregnancy, she visited our hospital and recommended to perform routine B‐ultrasound test. We found foetal hydrocephalus and extraventricular cysts with less amniotic fluid (AFI: 6.8 cm). The pregnancy was terminated and it was a female foetus. In 2008, her second pregnancy was completely uneventful and she has given birth to a normal baby girl. In 2009, during her third pregnancy, she again visited our hospital and recommended to perform routine B‐ultrasound test. That time, we found a small amount of liquid at the dark area in the foetus with very less amniotic fluid (AFI: 1.0 cm). The pregnancy was terminated and the gender of the foetus was unknown. In 2010, during her fourth pregnancy, we again recommended her to perform the routine B‐ultrasound test and we identified the foetus with lateral ventricle enlargement, abnormal structure of the skull, wide range of liquid dark areas, the structure of the brain midline became thinner. We also found that the foetal neck with an echo‐free zone, the range was 5.4 × 6 cm, showing clearly the separation inside with very less amniotic fluid (AFI: 1.9 cm). The pregnancy was terminated and it was a male foetus. In 2013, her fifth pregnancy was completely uneventful and she has given birth of a normal baby girl.

**Figure 3:**
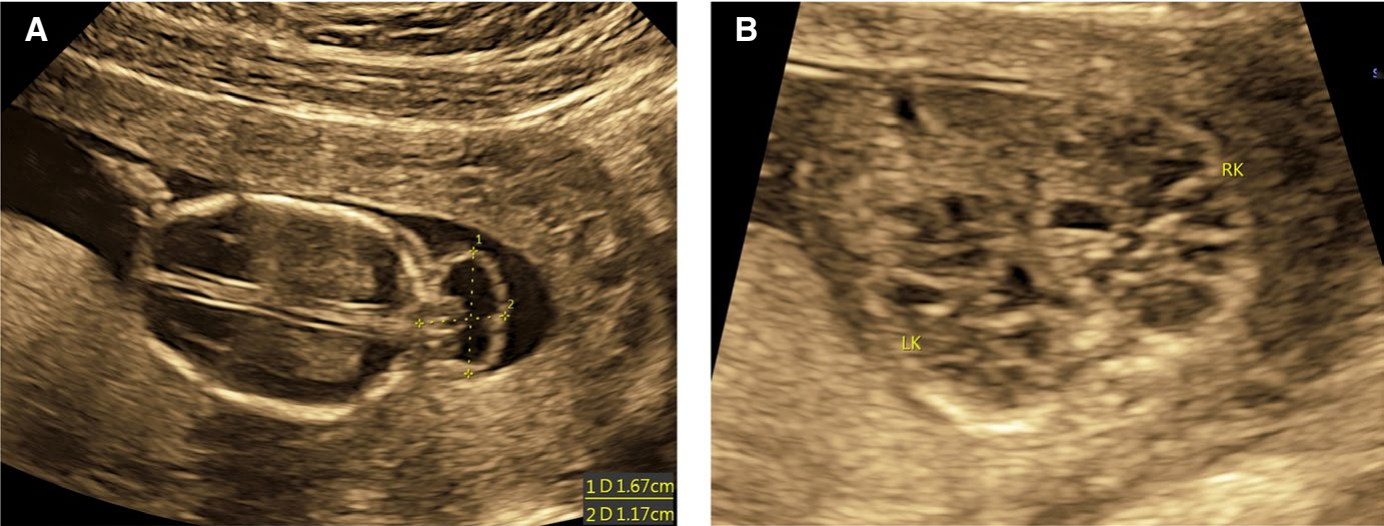
A and B, Ultrasonography examination revealed that bone defect in the posterior region of the calvarium with herniation of the brain (approximate 1.56 × 1.2 cm occipital encephalocele); bilateral polycystic kidneys (occupying the entire fatal abdomen)

In 2017, during her sixth pregnancy, she again visited our hospital and recommended to perform routine B‐ultrasound test. We found that the continuous echo of the skull in the occipital region was interrupted, about 0.96 cm wide. The cerebellum tissue bulged out and located in the extracranial cavity, forming a mass of about 1.56 × 1.2 cm, and the cranial cavity was significantly smaller (Figure 3A). The foetal kidneys were obviously enlarged, filled the entire abdominal cavity (Figure 3B). The size of the left kidney was about 3.0 × 2.1 × 1.9 cm, and the size of the right kidney was about 3.0 × 1.8 × 2.0 cm. There are many cystic echo regions of different sizes and shapes. Prenatal genetic diagnosis with amniocentesis at 18 weeks of gestation was performed.

### 3.2 Karyotype and chromosomal microarray analyses

Karyotype analysis found no chromosomal structural abnormality in the foetus (46, XX). Chromosomal microarray did not identify any pathogenic copy number variations (CNVs) in the chromosomes of the foetus.

After identifying no abnormality in both karyotype and chromosome microarray, and based on both the multiple similar adverse pregnancy history and clinical symptoms (foetal meningocele during pregnancy and bilateral polycystic Kidney with poor prognosis) of the foetus, the pregnant woman decided to terminate the pregnancy and perform further genetic molecular diagnosis to understand the underlying cause of the disease phenotype.

**Figure 4:**
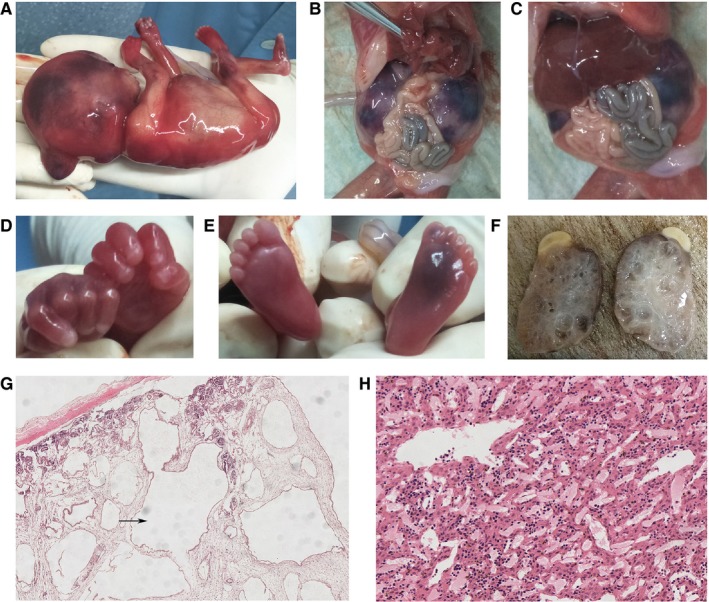
A‐F. A, Occipital encephalocele of the foetus. B and C, bilateral polycystic kidneys (occupying the entire fatal abdomen). D and E, No postaxial polydactyly. F, Bilateral grossly enlarged kidneys interspersed with small, pinhead‐sized cysts. G and H, Histology analysis of kidney and liver. G, Histology examination of kidney found that the presence of cysts in the kidney with thinning of renal cortex and glomerular atrophy. H, Histology examination of liver showed no abnormality

We observe the aborted foetus and found occipital encephalocele (Figure 4A), bilateral polycystic kidneys (occupying the entire fatal abdomen) (Figure 4B,C). We have not found postaxial polydactyly in both hand and feet of the foetus (Figure 4D,E). We also identified bilateral grossly enlarged kidneys interspersed with small, pinhead‐sized cysts (Figure 4F). Histology of kidney found cysts in kidney and thinning of renal cortex with glomerular atrophy (Figure 4G). Histology of liver showed no abnormality (Figure 4H).

Foetal skin tissue was taken for the extraction of genomic DNA. Whole exome sequencing has been done with foetal genomic DNA.

### 3.3 Whole exome sequencing and Sanger sequencing identified a homozygous novel variant in *CEP290*

**Figure 5:**
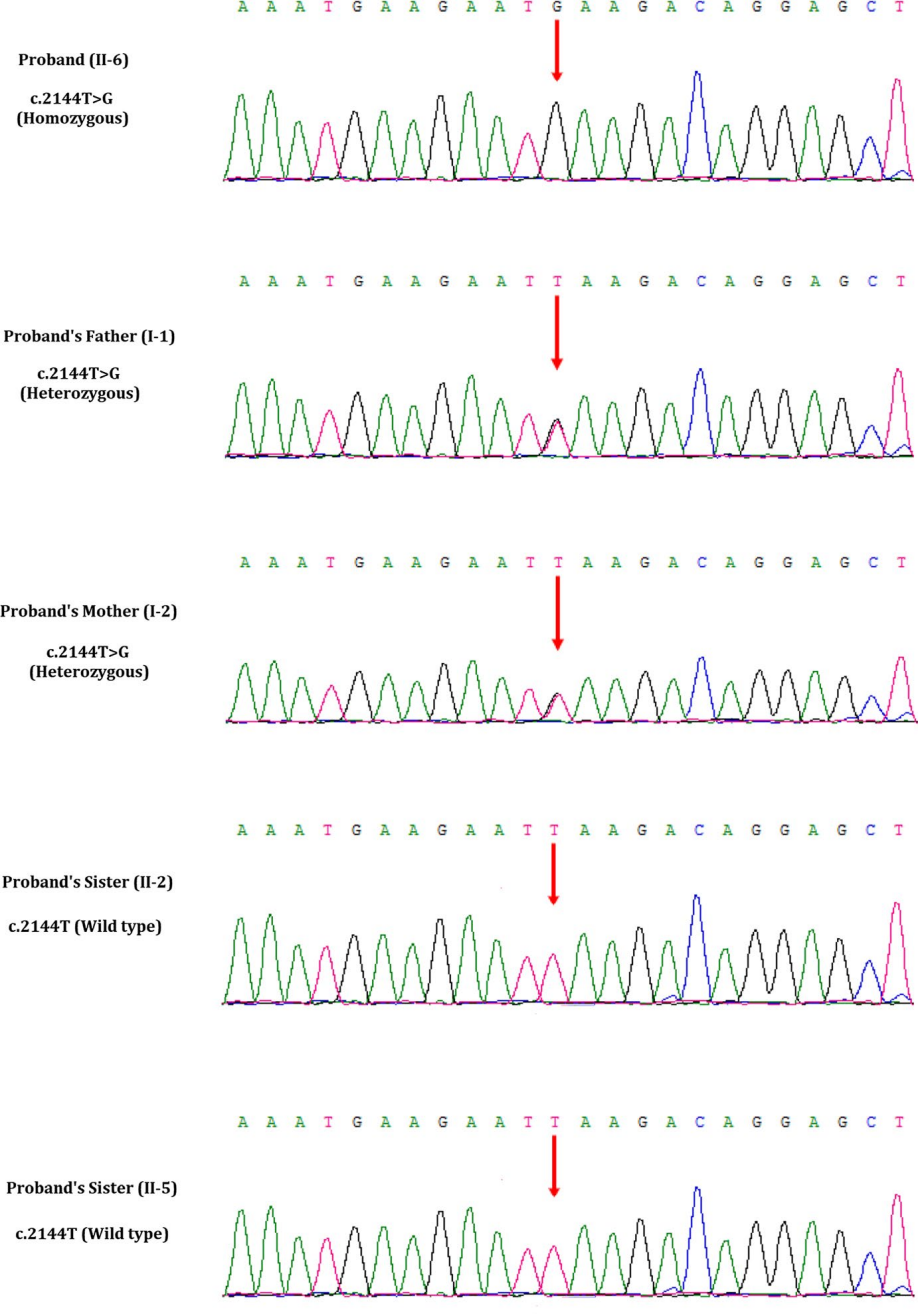
Partial DNA sequences in the *CEP290* gene by Sanger sequencing of the family. The reference sequence NM_025114.3 of *CEP290* gene was used

We performed whole exome sequencing of DNA from the skin of the foetus. Whole exome sequencing identified a novel homozygous variant (c.2144T>G, p.Leu715^*^) in the exon 21 of the *CEP290* gene in the foetus. This novel homozygous variant leads to a premature termination of translation which finally results into the formation of a truncated CEP290 protein of 714 amino acids instead of the wild‐type CEP290 protein consisting of 2479 amino acids. Hence, it is a *loss‐of‐function* variant. Sanger sequencing confirmed that both the father and mother of the foetus were harbouring this variant in a heterozygous state (Figure 5). Sanger sequencing also revealed that two healthy and phenotypically normal elder sisters (II‐2 and II‐5) of the foetus also lack of this variant. This variant is not found in 100 normal control individuals. This variant is also not present in the Human Gene Variant database (HGMD, http://www.hgmd.cf.ac.uk/), Online Mendelian Inheritance in Man (MIM, (https://www.omim.org). This homozygous novel variant is also not found in BGI's database, consisting of ~50 000 Chinese Han samples. We also did not find this variant in ExAC (exac.broadinstitute.org), gnomAD (https://gnomad.broadinstitute.org), dbSNP (https://www.ncbi.nlm.nih.gov/SNP) and1000 Genome Database (http://www.internationalgenome.org).

### 3.4 In vitro functional analysis and characterization of the novel variant by western blot

**Figure 6:**
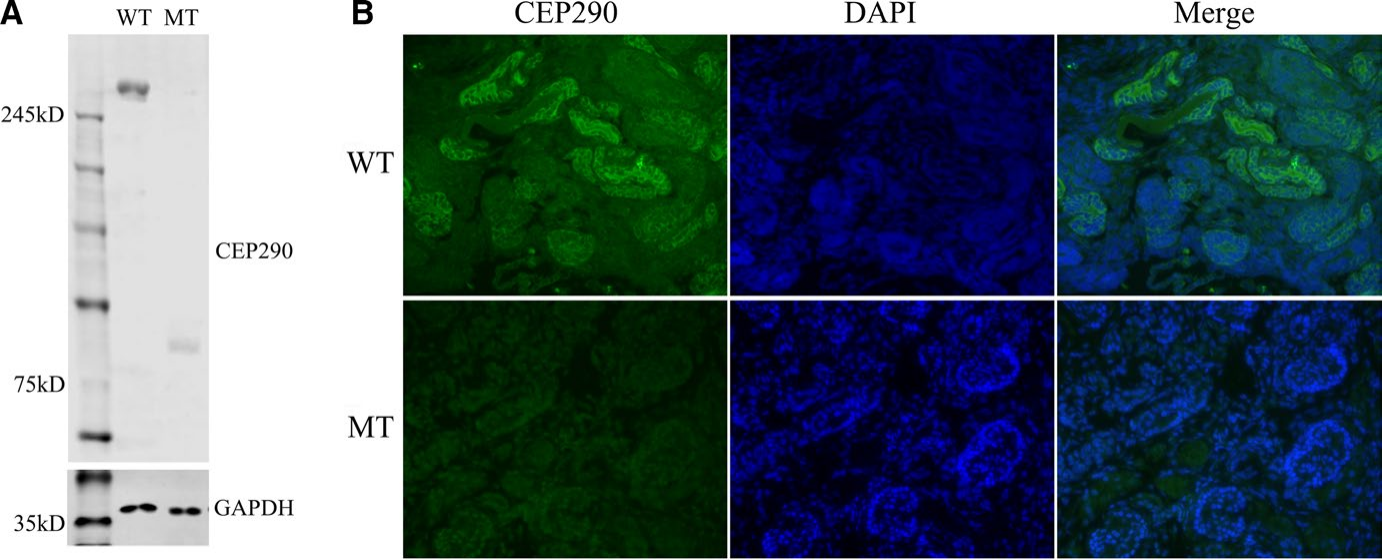
A, Protein expression analysis using Western blotting of kidney tissue obtained from patient MKS. Lane 1 showed the CEP290 (290 KD) expression in normal kidney tissues (wild‐type, WT). Lane 2 showed CEP290 expression in foetal kidney tissue (mutant type, MT). GAPDH levels, served as controls. B, Localization of CEP290 in kidney tissues of MKS patient and normal kidney tissue. DAPI, for nuclear staining (blue); CEP290, Anti‐ CEP290 antibody followed by an Alexa fluo488‐conjugated secondary antibody (green); Merge, DAPI nuclear staining plus anti‐CEP290 antibody. These images were observed using immunofluorescence microscopy (40×)

Western blot analysis showed that this variant (c.2144T>G, p.Leu715*) leads to the formation of a truncated CEP290 protein with the molecular weight of 84 KD compared with the wild‐type CEP290 protein of 290 KD. In addition, the expression of the mutated CEP290 protein in foetal kidney tissue was also significantly lower in comparison with the expression of the wild‐type CEP290 protein in normal kidney tissues (Figure 6A).

### 3.5 Immunofluorescence study of human kidney tissues

Immunofluorescence study showed that the expression of mutated *CEP290* was quite lower in foetal polycystic kidney tissue in comparison with the expression of wild‐type CEP290 protein in normal kidney tissue, which was consistent with the results of Western blot. In addition, we also observed that the localization of the mutated CEP290 protein in foetal kidney tissues, compared to the wild‐type CEP290 protein in normal kidney tissues with a magnification of 40× (Figure 6B).

**Figure 7:**
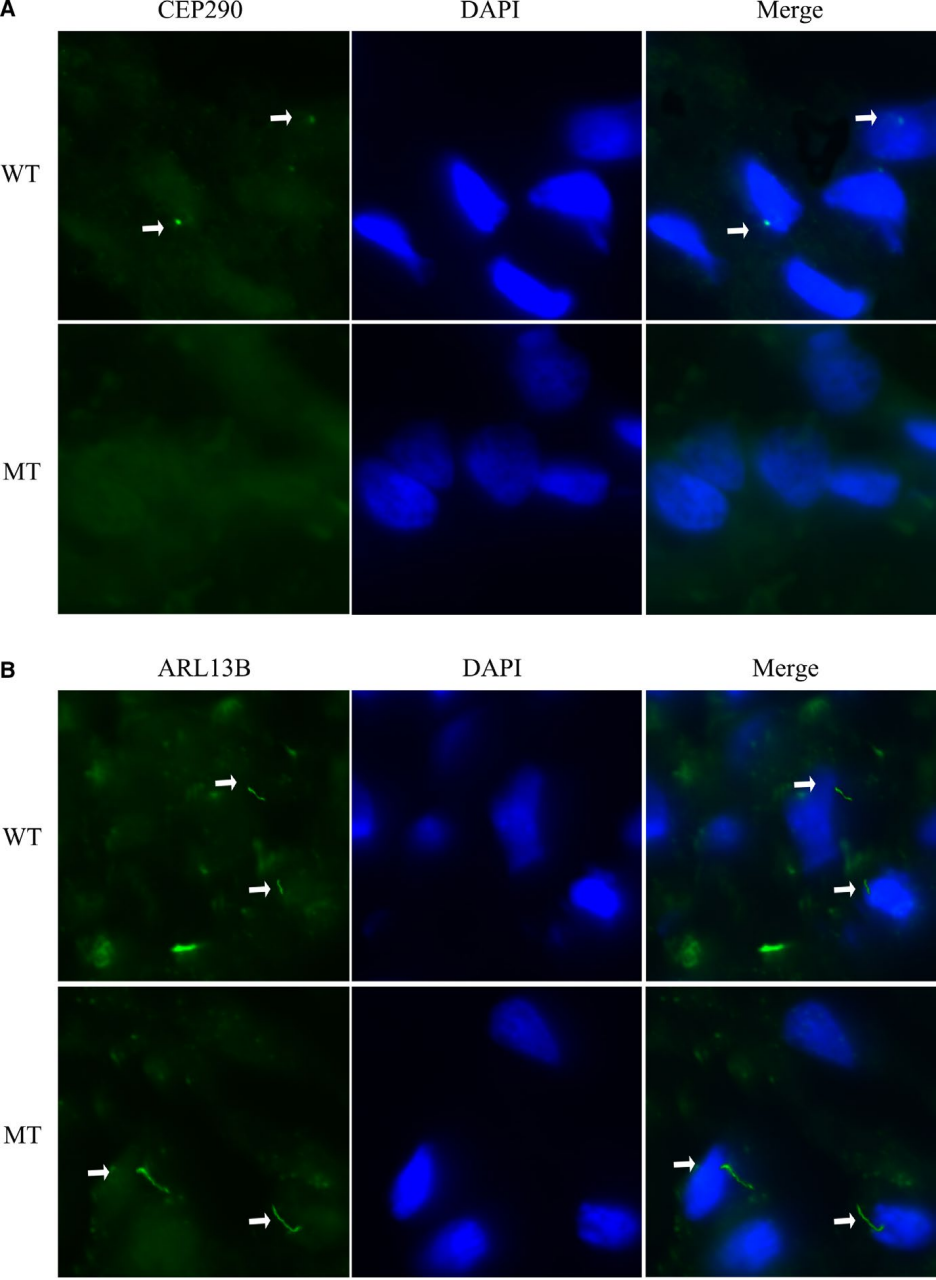
A, Localization of CEP290 in kidney tissues of MKS patient and normal kidney tissues (63×). DAPI, for nuclear staining (blue); CEP290, Anti‐CEP290 antibody followed by an Alexa Fluor 488‐conjugated secondary antibody (green); Merge, DAPI nuclear staining plus anti‐CEP290 antibody. B, Localization of ARL13B in kidney tissues of MKS patient and normal kidney tissues (63×). DAPI, for nuclear staining (blue); ARL13B, Anti‐ARL13B antibody followed by an Alexa Fluor 488‐conjugated secondary antibody (green); Merge, DAPI nuclear staining plus anti‐ARL13B antibody. The white arrow indicates the cilia. These images were observed using immunofluorescence microscopy (63×)

In order to understand the specific location of both wild‐type and mutated CEP290 in normal as well as in foetal kidney tissues, we used high magnification (63×) immunofluorescence imaging. In accordance with previous studies, Figure 7A showed CEP290 localization to the ciliary base in normal kidney tissue.^12^, ^13^ However, CEP290 had almost no expression in foetal polycystic kidney tissue. In order to observe the effect of CEP290 variant on ciliary structure, we detected the localization and expression of ARL13B (ADP‐ribosylation factor‐like protein 13B) in both normal and foetal polycystic kidney tissues by immunofluorescence assay. The expression of ARL13B in foetal polycystic kidney tissues with mutated CEP290 protein was similar to the normal kidney tissues with wild‐type CEP290 protein (Figure 7B). We also found difference in the length of cilia between the wild‐type and mutant kidney tissues. The mutant cilium appears longer in length than that of the wild‐type cilium.

### 3.6 In silico analysis

The variant (c.2144T>G, p.Leu715*) was predicted “disease causing” by Mutation Taster (http://mutationtaster.org/).^11^

## 4 DISCUSSION

In our present study, we described a 35‐years‐old pregnant Chinese woman from a non‐consanguineous Chinese family. She had a history of adverse pregnancy. During her sixth pregnancy, we found that the foetus was presented with occipital meningoencephalocele with a massively malformed brain, cystic dysplastic kidneys and the pregnant woman decided to terminate the pregnancy. We did not find any chromosomal abnormalities in the foetus by performing karyotype and chromosome microarray analysis. Then, we performed whole exome sequencing with the foetal DNA extracted directly from the foetal skin. Whole exome sequencing identified a novel homozygous variant (c.2144T>G, p.Leu715*) in the exon 21 of the *CEP290* gene. Sanger sequencing confirmed that both the father and mother of the foetus were harbouring this variant in a heterozygous state. According to the variant interpretation guidelines of American College of Medical Genetics and Genomics (ACMG), this variant is categorized as *“likely pathogenic”* variant.^10^

In vitro functional analysis of this variant (c.2144T>G, p.Leu715*) showed that this variant leads to the formation of a truncated CEP290 protein with the molecular weight of 84 KD with significantly lower expression level in foetal kidney tissue compared with the expression of the wild‐type CEP290 protein in normal kidney tissues (Figure 6A). Additionally, we also observed that the wild‐type CEP290 protein is localized into the ciliary base in normal kidney tissue while we found no expression of mutated CEP290 protein in foetal polycystic kidney tissue (Figure 7A). However, the expression of ARL13B in foetal kidney tissue with mutated CEP290 protein was similar to the normal kidney tissues with wild‐type CEP290 protein (Figure 7B).

In addition, here, we observed differences in the length of cilia between wild‐type and mutant kidney tissues. The length of the mutant cilium has found longer than that of the wild‐type cilium. However, structural defects of cilia as well as differences in ciliary length are correlated with severe developmental diseases.^14^ Polycystic kidney tissues of foetus have been suffering from MKS3 with mutation in TMEM67 gene showed longer cilia than wild‐type tissues.^15^ LCA patients with mutations in *CEP290* gene never been identified with an increased ciliary length. Hence, the structural defects of cilia as well as differences in ciliary length are tissue specific. In addition, regulation of the length of the primary cilia is very significant and dynamic process which is playing a key role in signal transduction through cilia.^16^ The structure and length of the cilia is very specific for its functional significance.^17^

Germline variants in *CEP290* gene rarely cause MKS4.^2^ Till now, all the MKS4 patients with *CEP290* gene variants were reported to be harbouring truncating variants in *CEP290*.^1^, ^2^, ^3^, ^18^, ^19^, ^20^, ^21^, ^22^, ^23^, ^24^, ^25^, ^26^ Among all the *CEP290* variants reported to cause MKS4 are majorly frameshift or non‐sense variants.^18^, ^19^, ^20^, ^21^, ^22^, ^23^, ^24^, ^25^, ^26^, ^27^ *CEP290* gene encodes the centrosomal protein CEP290 with 2479 amino acids.^28^ Centrosomal protein CEP290 showed a highly tissue specific expression, majorly in embryonic tissues, but not expressed in adult tissues or organs.^15^ Hence, the expression pattern of centrosomal protein CEP290 suggesting us that it is playing a significant role during embryonic development.^28^

Centrosomal protein CEP290 is playing a key role in both early and late steps in cilia formation.^2^ It may involve in the progressive loss of centriolar satellites and simultaneous formation of capped ciliary vesicles (CCVs) through the transition of primary ciliary vesicles (PCVs).^1^ It helps in recruiting RAB8A to the primary cilia and also targets the satellite proteins of centriole and transfer it to centrosome.^2^, ^29^ Sayer et al and Valente et al reported that CEP290 is localized into the cilia, centrosome and nucleus with a cell cycle‐dependent manner and playing a significant role in chromosomal segregation.^30^, ^31^ Likely kinetochore, centrosomal protein CEP290 also consisting of evolutionarily highly conserved domains and motifs which are suggesting that these domains and motifs have a specific function for domain assembly within CEP290.^32^ There are 13 coiled‐coil domains in centrosomal protein CEP290 which includes chromosome segregation ATPase, kinase inducible domain, tropomysin homology domain, nuclear localization signalling domain and ATP/GTP binding domain.^33^ CEP290 protein also involved in N‐glycosylation, phosphorylation, tyrosine sulfation, amidation and N‐myristoylation.^34^ Hence, germline variants in CEP290 may adversely affect on the intraciliary and axonal transport, followed by loss of axon guidance and outgrowth which may explain the malformation of brain observed in patients with MKS4.

In our present study, we identified a 17+1‐week pregnant Chinese female with a history of adverse pregnancy. During her sixth pregnancy, she decided to terminate her pregnancy because of ultrasonographic features of the foetus. The foetus was identified with all classic clinical symptoms of MKS, that is occipital meningoencephalocele, enlarged cystic dysplastic kidneys, but without postaxial polydactyly and hepatobiliary ductal plate malformation. Whole exome sequencing identified a novel homozygous *loss‐of‐function* variant in *CEP290* gene in the foetus. Both the father and mother of the foetus ware harbouring the same variant in a heterozygous state. Although the inheritance pattern of the candidate variant in this family is quite resembling with the usual inheritance pattern of MKS in consanguineous families, but this studied family is truly non‐consanguineous. Interestingly, MKS4 with the variant in *CEP290* gene is more prevalent in consanguineous families.^25^ Our present case is the first report of *CEP290* associated MKS4 in Chinese population.

Meckel syndrome is a very rare and extremely heterogenous (in both genotypically and phenotypically) disorder. So, single gene sequencing or targeted next‐generation sequencing is not always identified the candidate variants in patients with MKS. Therefore, whole exome sequencing is more accurate and reliable for identifying the candidate gene and variants underlying the disease phenotype in patients with MKS. Whole exome sequencing (WES) is one of the most significant technologies for identifying candidate gene and disease‐causing variants in patients with MKS. Whole exome sequencing is more accurate, rapid and cost‐effective tool for early and timely molecular genetic analysis allowing clinicians for making accurate clinical diagnosis.^35^, ^36^ So, our present study not only report the first variant of *CEP290* gene in a patient with MKS4 in Chinese population, but also strongly emphasizes the significance of WES as an accurate, rapid and cost‐effective tool for molecular genetic analysis for the patients with MKS.

## CONFLICT OF INTEREST

The authors confirm that there are no conflicts of interest.

## AUTHOR CONTRIBUTIONS

SB, YZ and RZ designed the study. RZ, SC, FC, ZM, X.H and RS conducted acquisition and analysis of all the clinical data. PH, SK, YL and YG made WES pipeline and analysed the data. JL, ZW and ZY selected patients and performed WES. SB, YZ, RZ, CL and SKD supervised manuscript preparation and edited the manuscript.

## Data Availability

All data used for the analyses in this report are available in the CNGB Nucleotide Sequence Archive (CNSA: https://db.cngb.org/cnsa). Accession number: CNP0000361.
